# Intrameatal Treatment

**Published:** 1909-03-20

**Authors:** F. Faulder White


### EDITOR'S LETTER-BOX.

### INTRAMEATAL TREATMENT.

*To the Editor of* The Hospital.

Sir,—With reference to the criticism of my little book, "Infected Ears," I should like to say that, though the value of irrigation and intrameatal operative treatment may now be admitted by many aural surgeons, it is certainly true that, for a considerable period, any advocacy of an alternative treatment to the radical mastoid operation met with something approaching contempt. To my mind it is a matter of importance that the fact, still disputed by some, of the general efficacy of intrameatal treatment should be demonstrated, for in its acceptance lies the chief hope for a multitude of sufferers from infected ears.

I am, yours, etc.,

F. Faulder White, F.R.C.S.

